# Comparative Analysis of Tools and Approaches for Source Tracking *Listeria monocytogenes* in a Food Facility Using Whole-Genome Sequence Data

**DOI:** 10.3389/fmicb.2019.00947

**Published:** 2019-05-09

**Authors:** Balamurugan Jagadeesan, Leen Baert, Martin Wiedmann, Renato H. Orsi

**Affiliations:** ^1^Nestlé Institute of Food Safety and Analytical Sciences, Nestlé Research, Lausanne, Switzerland; ^2^Department of Food Science, Cornell University, Ithaca, NY, United States

**Keywords:** *Listeria monocytogenes* (*L. monocytogenes*), whole genome sequence (WGS), high quality single nucleotide polymorphism (hqSNP), whole genome MLST (wgMLST), core genome MLST (cgMLST), CFSAN pipeline, Lyve-SET, smoked salmon

## Abstract

As WGS is increasingly used by food industry to characterize pathogen isolates, users are challenged by the variety of analysis approaches available, ranging from methods that require extensive bioinformatics expertise to commercial software packages. This study aimed to assess the impact of analysis pipelines (i.e., different hqSNP pipelines, a cg/wgMLST pipeline) and the reference genome selection on analysis results (i.e., hqSNP and allelic differences as well as tree topologies) and conclusion drawn. For these comparisons, whole genome sequences were obtained for 40 *Listeria monocytogenes* isolates collected over 18 years from a cold-smoked salmon facility and 2 other isolates obtained from different facilities as part of academic research activities; WGS data were analyzed with three hqSNP pipelines and two MLST pipelines. After initial clustering using a k-mer based approach, hqSNP pipelines were run using two types of reference genomes: (i) closely related closed genomes (“closed references”) and (ii) high-quality *de novo* assemblies of the dataset isolates (“draft references”). All hqSNP pipelines identified similar hqSNP difference ranges among isolates in a given cluster; use of different reference genomes showed minimal impacts on hqSNP differences identified between isolate pairs. Allelic differences obtained by wgMLST showed similar ranges as hqSNP differences among isolates in a given cluster; cgMLST consistently showed fewer differences than wgMLST. However, phylogenetic trees and dendrograms, obtained based on hqSNP and cg/wgMLST data, did show some incongruences, typically linked to clades supported by low bootstrap values in the trees. When a hqSNP cutoff was used to classify isolates as “related” or “unrelated,” use of different pipelines yielded a considerable number of discordances; this finding supports that cut-off values are valuable to provide a starting point for an investigation, but supporting and epidemiological evidence should be used to interpret WGS data. Overall, our data suggest that cgMLST-based data analyses provide for appropriate subtype differentiation and can be used without the need for preliminary data analyses (e.g., k-mer based clustering) or external closed reference genomes, simplifying data analyses needs. hqSNP or wgMLST analyses can be performed on the isolate clusters identified by cgMLST to increase the precision on determining the genomic similarity between isolates.

## Introduction

*Listeria monocytogenes* is a major problem for the food industry and food business operators, including small manufacturers and retailers, as this organism is ubiquitous in nature and is highly adapted to conditions usually used for food preservation and safety (Buchanan et al., 2017). *L. monocytogenes* has been shown to be able to persist for more than 10 years in food facility settings (Orsi et al., 2008; Carpentier and Cerf, 2011) where the organism can re-contaminate the final product multiple times. Hence, of great relevance to the food industry is the ability to differentiate persistent strains from transient strains. Persistence of a *L. monocytogenes* strain in a food facility can often be traced back to unhygienic design of equipment, infra-structure problems (e.g., hollow framework, cracks on floor) or inefficient cleaning and sanitation procedures. While some authors (Bridier et al., 2015; Nowak et al., 2017) propose biofilm formation as a key mechanism for persistence, there appears to be limited evidence that *L. monocytogenes* forms true biofilms (Ferreira et al., 2014) and persistence may be more likely due to survival in niches that are not reached by the cleaning and sanitation practices used in a given facility. High prevalence of transient strains may on the other hand indicate that *L. monocytogenes* are being introduced repeatedly from outside sources. Different measures must be taken in each case and significant financial costs are expected to be necessary to resolve the problem. In addition to identifying persistent strains, the food industry also has a need to investigate the relatedness of organisms involved in a single contamination event to aid accurate root cause analysis. Findings of such an analysis can optimize the implementation of appropriate corrective actions to prevent recurrence of contamination issues.

Molecular subtyping tools can provide the information needed to study the relatedness between isolates and to discriminate between persistent and transient strains by differentiating them at the sub-species level. While other subtyping methods have been used in the past (e.g., ribotyping), particularly by industry, for many years, pulsed field gel electrophoresis (PFGE) has been the gold standard subtyping tool for *L. monocytogenes*. PFGE has been used not only for source tracking of *L. monocytogenes* at food settings, but also in outbreak investigations throughout the globe (Swaminathan et al., 2001). However, the advent of the next-generation sequencing (NGS) technology has shifted the paradigm toward the application of whole genome sequencing (WGS) as the most specific molecular tool for subtyping. The high-throughput capability and the increased speed of NGS followed by the steep drop in costs and the development of new bioinformatics tools to process the data has allowed WGS analysis to become the current subtyping method of choice for *L. monocytogenes* outbreak investigation in the United States (Allard et al., 2016; Jackson et al., 2016), France (Moura et al., 2017), and Denmark (Kvistholm Jensen et al., 2016) among other countries.

There are two main bioinformatics approaches that can be used for subtyping and clustering of bacterial organisms based on genomic variations: (i) assessment of single nucleotide differences based on high quality single nucleotide polymorphisms (hqSNPs) within the whole genome following initial clustering of the isolates using a reference-free SNP-based approach; and (ii) assessment of allelic differences based on comparison of a defined selection of genes (cgMLST and wgMLST). In the United States, the Food and Drug Administration (FDA) has developed and applied the CFSAN hqSNP pipeline (Davis et al., 2015) for subtyping of foodborne pathogens, such as *L. monocytogenes*. This pipeline has been validated and applied in several investigations on the food source of an outbreak. The public health agency in the United States, the Centers for Disease Control and Prevention (CDC) has developed and applied the Lyve-SET hqSNP pipeline (Katz et al., 2017), which has also been validated and used in investigations of foodborne disease outbreaks caused by *L. monocytogenes* and other foodborne pathogens (Jackson et al., 2016). While these two hqSNP pipelines require skills to work in a command-line, Linux-based environment, a hqSNP pipeline available from BioNumerics (hereafter designated as BN pipeline) provides a graphical interface solution while keeping similar functionalities that can be used by end-users (microbiologists) with limited bioinformatics expertise. All of these hqSNP pipelines can theoretically identify all the single nucleotide polymorphisms present in a genome. In addition, parameter settings in both the CFSAN and the Lyve-SET pipelines can be used to prevent assessing SNP differences that fall within horizontally transferred elements such as prophage sequences, transposons, and plasmids, which can obscure the isolates’ true phylogeny (Ochman and Jones, 2000; Galtier, 2007). In specific cases when further discrimination of the isolates may be required, identification and analysis of prophages and other horizontally transferred elements may be useful (Knabel et al., 2012). All three hqSNP pipelines (i.e., CFSAN, Lyve-SET, and BioNumerics) are specifically designed to assess the differences among closely related isolates (Davis et al., 2015; Chen et al., 2017; Katz et al., 2017) since the analysis relies upon a closely related reference genome. To overcome the limitation of assessing only closely related isolates and the dependency on a reference genome, a genome-wide multi locus sequence typing (MLST) approach has been developed and applied. In France, the Institut Pasteur has developed and validated an allelic-based subtyping scheme based on the core genome of hundreds of *L. monocytogenes* genomes available (Moura et al., 2016). This cgMLST scheme, which includes 1748 genes, has been implemented in the BIGSdb software (Jolley and Maiden, 2010) to provide online access to analysis (Moura et al., 2016) and has been used by governmental agencies for real-time surveillance involving thousands of *L. monocytogenes* genomes (Moura et al., 2017; ECDC-EFSA, 2018). This approach has the advantage of not relying on a reference genome, which makes the results comparable between laboratories. In addition, the cgMLST scheme can be used for comparison of any *L. monocytogenes*, regardless of their relatedness level. However, cgMLST has the disadvantage of only assessing differences present within the core genome of *L. monocytogenes*, which represents ∼58% of the genome in terms of number of genes and ∼54% in terms of the length of the genome (Moura et al., 2016). Hence, differences not present in the core genome (e.g., polymorphisms present in intergenic regions or in accessory genes) are not assessed. Another genome-wide MLST scheme being used by the US CDC and PulseNet International is known as the whole genome MLST (wgMLST) pipeline provided through BioNumerics (Jackson et al., 2016; Nadon et al., 2017). This proprietary scheme has the same advantages described for the cgMLST scheme without the disadvantage of assessing only part of the protein-coding genes in the genomes. Nevertheless, the wgMLST scheme may still miss some polymorphisms, such as those found in intergenic regions or in very rare accessory genes that are not part of the scheme. In addition, both wgMLST and cgMLST analysis standard results provide no information regarding the nucleotide changes associated with the allelic differences observed; while clustering algorithms have been used to construct phylogenetic trees from allelic difference matrices, use of cladistic methods (i.e., parsimony or likelihood methods) is only possible with sequence data. Both cgMLST and wgMLST pipelines are faster and provide results that are more standardized and comparable than the hqSNP pipelines. This allows for easier exchange of results between laboratories and others, which may be crucial in time-sensitive cases and when non-specialists are involved in data interpretation.

In this study, we assessed the impact of the choice of hqSNP or genome-wide MLST (i.e., cgMLST and wgMLST) pipeline on interpretation, using a longitudinal set of 40 *L. monocytogenes* isolates as a model system. The aim was not to optimize the parameters for analyses by different approaches (SNP and cg/wgMLST), but rather, apply the default settings as those are the settings most likely to be used by many industry microbiologists and to determine whether this standardized approach could allow for a meaningful analyses and interpretation of the data. The 40 isolates used in the study were collected over a period of 18 years from a cold-smoked salmon food processing facility as part of academic research projects (Norton et al., 2001b; Hoffman et al., 2003; Lappi et al., 2004; Thimothe et al., 2004; Hu et al., 2006; Malley et al., 2013). Results obtained with the CFSAN, Lyve-SET and BN hqSNP pipelines were compared among each other and were also compared to results obtained with cgMLST and wgMLST analyses in BioNumerics. We specifically assessed (i) the impact on interpretation of the hqSNP pipelines when different reference sequences were used, (ii) the ability of each hqSNP and MLST pipeline to differentiate and cluster the isolates by comparing tree and dendrogram topologies derived from hqSNP matrices and allelic difference matrices, and (iii) the impact of pairwise comparison of isolates using hqSNP and allelic differences on results inference. The data obtained were also used to assess whether isolates in our dataset represent persistent or transient strains (defined here as a genetically distinct *L. monocytogenes* that could be represented by a group of genetically closely related isolates) (van Belkum et al., 2007).

## Materials and Methods

### Isolate Selection

Food Microbe Tracker (Vangay et al., 2013) was used to search for isolates belonging to ribotype DUP 1062 (A, B, C, or D variants) and collected from a single cold-smoked salmon food processing facility, which had been sampled between 1998 and 2015 for a longitudinal analysis of persistence in this facility (Norton et al., 2001b; Hoffman et al., 2003; Lappi et al., 2004; Thimothe et al., 2004; Hu et al., 2006; Malley et al., 2013). The search, carried out on September 11, 2017, resulted in 57 isolates matching the above-mentioned criteria. Out of those 57 isolates, 44 were from the facility’s environment, 8 were from foods, and the remaining 5 did not have details of the sample type or location available. Six were isolated in 1998, 20 in 2000, 5 in 2001, 1 in 2002, 1 in 2004, 4 in 2007, 2 in 2009, 13 in 2011, 3 in 2012 and 3 in 2015. In order to balance our set, we selected all 25 isolates from 1998, 2001, 2002, 2004, 2007, 2009, 2012, 2015, 8 isolates from 2000, and 7 isolates from 2011. Isolates from 2000 were distributed across five different months; February (*n* = 3), March (*n* = 7), July (*n* = 7), August (*n* = 2) and September (*n* = 1). February and March isolates were combined into group 2000A (*n* = 10) while isolates from July, August and September were combined into group 2000B (*n* = 10). Four isolates were then selected from each group such that none of the isolates shared the same ribotype variant and sampling month and same specific source (e.g., drain 18, west coast fish). Likewise, isolates from 2011 were distributed across 6 months; May (*n* = 3), June (*n* = 2), July (*n* = 2), September (*n* = 3), November (*n* = 1) and December (*n* = 1). Isolates from May, June and July were combined into group 2011A (*n* = 7) and isolates from September, November, and December were combined into group 2011B (*n* = 5). Four isolates were selected from group 2011A and three isolates were selected from group 2011B, following the same criteria applied for selection of isolates from groups 2000A and 2000B. Therefore, the final dataset contained 40 ribotype DUP-1062 isolates sampled from the same food facility between 1998 and 2015. Two additional isolates collected in 1998 and ribotyped as DUP-1062 but isolated from two different food facilities were added for comparison for a total of 42 isolates that were whole-genome sequenced (Table 1).

**Table 1:** *Listeria monocytogenes*^a^ isolated over a period of 18 years from a cold-smoked salmon facility along with two additional isolates from two different facilities.

Isolate	Source	Specific source	Year	Month	Ribotype	MLST ST^b^
FSL N1-0013^c^	Food	Whitefish	1998	May	DUP-1062	ST121
FSL N1-0051^c^	Environment	Equipment	1998	August	DUP-1062	ST321
FSL N1-0053	Environment	Drain, raw area	1998	August	DUP-1062	ST321
FSL N1-0110	Food	Salmon brine	1998	August	DUP-1062A	ST321
FSL N1-0254	Environment	Drain	1998	October	DUP-1062A	ST321
FSL N1-0255	Environment	NA	1998	October	DUP-1062A	ST321
FSL N1-0256	Environment	Drain	1998	October	DUP-1062A	ST321
FSL N1-0400	Environment	Drain	1998	October	DUP-1062A	ST321
FSL H1-0081	Environment	Crates	2000	February	DUP-1062A	ST321
FSL H1-0159	Environment	Drain 54	2000	March	DUP-1062C	ST371
FSL H1-0193	Environment	Drain 18	2000	March	DUP-1062A	ST321
FSL H1-0221	Environment	Door handle	2000	February	DUP-1062A	ST321
FSL H1-0258	Food	West coast	2000	July	DUP-1062A	ST321
FSL H1-0322	Environment	Drain 54	2000	July	DUP-1062C	ST371
FSL H1-0328	Environment	Cold smoking-room floor	2000	July	DUP-1062A	ST321
FSL H1-0506	Food	West coast	2000	August	DUP-1062D	ST121
FSL T1-0027	Environment	Floor	2001	March	DUP-1062A	ST321
FSL T1-0029	Environment	Apron	2001	March	DUP-1062A	ST321
FSL T1-0077	Environment	Apron	2001	March	DUP-1062A	ST321
FSL T1-0261	Food	Norwegian Salmon	2001	July	DUP-1062A	ST321
FSL T1-0938	Environment	White tubs	2001	November	DUP-1062A	ST321
FSL L4-0166	Environment	Drain near filet table	2002	October	DUP-1062A	ST321
FSL H6-0175	Environment	Cutting board, trimming area	2004	June	DUP-1062A	ST321
FSL R6-0665	Food	RTE product	2007	September	DUP-1062C	ST121
FSL R6-0670	Environment	Hand truck (dolly)	2007	September	DUP-1062C	ST121
FSL R6-0682	Environment	After skinning machine	2007	September	DUP-1062C	ST121
FSL R6-0909	Environment	Drain 15	2007	December	DUP-1062A	ST321
FSL V1-0034	Environment	Drain 2	2009	February	DUP-1062A	ST321
FSL V1-0142	Environment	Drain 4	2009	October	DUP-1062A	ST321
FSL M6-0150	Environment	Cutting table drain	2011	May	DUP-1062A	ST321
FSL M6-0204	Environment	Food contact surface	2011	May	DUP-1062D	ST121
FSL M6-0296	Environment	Drain in cooler room 9	2011	June	DUP-1062D	ST321
FSL M6-0306	Environment	Drain in cooler room 8	2011	June	DUP-1062A	ST321
FSL M6-0594	Environment	Drain in sturgeon room	2011	September	DUP-1062A	ST321
FSL M6-0755	Environment	Drain in sturgeon room	2011	November	DUP-1062A	ST321
FSL M6-0810	Environment	Drain in sturgeon room	2011	December	DUP-1062A	ST321
FSL M6-0958	Environment	Drain in sturgeon room	2012	February	DUP-1062A	ST321
FSL M6-1133	Environment	NA	2012	November	DUP-1062A	ST321
FSL M6-1145	Environment	NA	2012	November	DUP-1062A	ST321
FSL R9-4003	NA	NA	2015	≤May^d^	DUP-1062A	ST199
FSL R9-4438	Environment	Door handle	2015	≤April^d^	DUP-1062A	ST321
FSL R9-4443	NA	NA	2015	≤May^d^	DUP-1062A	ST321

*^a^All 42 isolates belong to L. monocytogenes lineage II and serotype 1/2a. With the exception of FSL N1-0013 and FSL N1-0051, all other isolates were isolated from the same food facility. ^b^In silico 7-gene MLST Sequence Type. ^c^FSL N1-0013 and FSL N1-0051 were isolated from two different food facilities. ^d^Exact month of collection not available. NA, not available.*

### DNA Extraction and Sequencing

Isolates were streaked from −80°C glycerol cultures onto BHI plates and incubated at 37°C overnight (∼15 h-17 h). DNA was extracted using the same protocol previously described in Stasiewicz et al. (2015). High quality DNA at concentration ∼10 ng/μl was submitted for library preparation using the Nextera kit according to manufacturer’s instructions (Illumina, Inc., San Diego, CA, United States) and sequenced using the Illumina MiSeq instrument (Illumina, Inc., San Diego, CA, United States) at the Cornell University Veterinary Molecular Diagnostics Laboratory.

### *De novo* Assembly and Quality Controls

Low quality raw reads were trimmed off using Trimmomatic version 0.33 (Bolger et al., 2014) with the parameters: LEADING:3 TRAILING:3 SLIDINGWINDOW:4:15 MINLEN:36. Trimmed reads were assessed for overall quality using FASTQC v0.10.1^1^. Files given the “PASS” flag under “basic statistics,” “per base sequence quality,” and “per sequence quality scores” were considered “good quality” and were further used for analyses. Good quality data were then used to generate draft assemblies using SPAdes version 3.8.0 (Bankevich et al., 2012) with the following parameter values: -k 127 –careful. QUAST 2.4 (Gurevich et al., 2013) (with the parameter –min_contig 1 and other default parameters) was used to assess the qualities of the assemblies, such as the total length of the assemblies, N50, and the total number of contigs. Assemblies were considered of good quality if they had: (i) total length = 3.0 Mb ± 0.3 Mb; (ii) N50 greater than 20 Kb; and (iii) number of contigs lower than 450. BBmap version 35.49 (Bushnell, 2015) and samtools version 0.1.19-96b5f2294a (Li et al., 2009) were used to determine the average coverage for each genome as previously described (Carroll et al., 2017). An average coverage greater than 30× was considered acceptable.

### SNP Analysis

A k-mer based approach was initially used to assess the clustering of the 42 isolates. All 140 *L. monocytogenes* closed assemblies available on NCBI RefSeq database at the time (October 16, 2017) were downloaded to be included in this k-mer based clustering. The program kSNP3 (Gardner et al., 2015) was used to identify core pairwise SNPs between all 140 closed assemblies and the 42 draft assemblies generated in this study using a k-mer of size 17 (-k 17). The k-mer size of 17 nucleotides was chosen as this was the shortest k-mer that provided a uniqueness greater than 99% (k-mer 17 uniqueness = 99.5%; k-mer 15 uniqueness = 97.9%). A parsimony tree were generated by kSNP3 using the set of core SNPs identified. Based on these results, 41 out of the 42 isolates sequenced here were grouped into three distinct clusters (referred to as 1, 2, and 3). One isolate, FSL R9-4003, did not cluster with any other isolate from our study and, therefore, was not further used in the hqSNP analyses presented here.

### Selection of Reference Genomes for hqSNP Analysis

As hqSNP analysis requires use of a closely related reference genome, different reference genomes were used for each cluster. Here, we assessed the impact of using different reference genomes on the outcomes of the hqSNP analyses. For the three distinct clusters identified by kSNP3, three closed assemblies from NCBI RefSeq database were selected from each cluster to be used as references for a given cluster. These references are called “closed references.” Initial hqSNP analysis further separated cluster 3 into two distinct sub clusters (3a and 3b); hence, subsequent additional hqSNP analyses were conducted using reference genomes obtained from the genomes sequenced here. Separate reference genomes were selected among the 42 genomes sequenced here for clusters 1, 2, 3a, and 3b; the specific reference genome for each of these clusters/subclusters was selected based on a combination of high average coverage, high N50, high total genome size and low number of contigs. The chosen references had: (i) average coverage greater than 50×; (ii) N50 greater than 100,000; (iii) number of contigs lower than 120; and (iv) either had a total genome size greater than 3.1 MB or represented the largest genome size among genomes in a given cluster. Hence, isolates FSL N1-0013, FSL H1-0159, FSL T1-0027, and FSL T1-0077 were chosen as references for analysis within clusters 1, and 2, and sub-clusters 3a and 3b, respectively (Table 2), and are referred to as “draft references.”

**Table 2:** Selection of reference genomes (closed and draft references) for hqSNP analysis.

Cluster	Reference	Length	Average coverage	Contigs	N50
Cluster 1 closed	NZ_HG813249.1	3,072,826	150×	1	NA
Cluster 1 draft	FSL N1-0013	3,112,177	102×	27	462,476
Cluster 2 closed	NZ_CP019617.1	2,989,685	182×	1	NA
Cluster 2 draft	FSL H1-0159	3,034,949	87×	113	108,201
Cluster 3 closed	NZ_CP019623.1	2,940,913	181×	1	NA
Cluster 3a draft	FSL T1-0027	3,045,313	142×	54	235,624
Cluster 3b draft	FSL T1-0077	3,112,454	201×	31	586,189

*NA, not applicable.*

### CFSAN and Lyve-SET Pipeline

The CFSAN hqSNP pipeline v. 1.0.0 was run with the default settings. The default settings are used by FDA in event investigations (Burall et al., 2017; Chen et al., 2017) and includes all the controls to prevent identification of SNPs in repetitive regions and horizontally transferred regions (e.g., prophages). Use of default settings also allow for better compatibility across different versions of the pipeline. The Lyve-SET pipeline was run with the following parameter values: –allowed_Flanking 500 –mask-phages –mask-cliffs. Allowed_Flanking of 500 was chosen as it provides similar control on the number of SNPs per window size (less than two SNPs in a window of 500 nucleotides) as the CFSAN hqSNP pipeline default settings (less than four SNPs in 1,000 nucleotides). Mask_phages was used to specifically filter out SNPs falling in regions corresponding to prophage sequences. Mask-cliffs was chosen to prevent calling SNPs in repeat regions and regions with sequence anomalies (Katz et al., 2017). The same reference genomes (described in section “Selection of Reference Genomes for hqSNP Analysis” and Table 2) were used with both pipelines.

### BN hqSNP Pipeline

The hqSNP pipeline implemented in BioNumerics V7.6.2 (Applied Maths, Sint-Martens-Latem, Belgium) was run with default settings for read mapping against the reference genomes described above using Bowtie (minimum total coverage of 3, minimum forward coverage of 1, minimum reverse coverage of 1) (see section “Selection of Reference Genomes for hqSNP Analysis”). After mapping, hqSNP analysis was performed using the “Strict filtering” setting (minimum total coverage of 5, minimum forward coverage of 1, minimum reverse coverage of 1). The pairwise SNP matrix was determined by carrying out “cluster analysis” and selecting “categorical (differences).”

### cg/wgMLST and Sequence Typing

BioNumerics V7.6.2 (Applied Maths, Sint-Martens-Latem, Belgium) was used for the cg/wgMLST analysis. The *L. monocytogenes* wgMLST (4797 loci scheme; defined by Applied Maths, Sint-Martens-Latem, Belgium) and cgMLST (1748 loci scheme) (Moura et al., 2016) pipelines implemented in BioNumerics V7.6.2 (Applied Maths, Sint-Martens-Latem, Belgium) were used for the analyses. Assembly free and assembly based allele calling were carried out using the default settings. SPAdes version 3.7.1 was used for the *de novo* assembly. The allele differences were obtained by “cluster analysis” and selecting “categorical (differences).” Neighbor joining was used to make the dendrograms. Sequence types (ST) were assigned by BioNumerics V7.6.2 using the traditional seven housekeeping loci MLST scheme (Ragon et al., 2008) and sequence data for the isolates were extracted from their genome data.

### Comparison of Phylogenetic Trees and Dendrograms

Phylogenetic trees were built from hqSNP matrices and dendrograms from cg/wgMLST allelic differences. HqSNP matrices obtained from each hqSNP pipeline (i.e., CFSAN, Lyve-SET, and BN hqSNP) were used to generate 100× bootstrapped phylogenetic trees using RAxML version 8.2.4 (Stamatakis, 2014) and the following parameter values: -f a -p 23283 -x 13373 -N 100 -m GTRCATX. In order to assess whether distinct hqSNP or cg/wgMLST pipelines could result in different phylogenetic trees or dendrograms for the same dataset (cluster), bipartition trees (or dendrograms) for the same cluster but generated from distinct pipelines were pairwise compared using the Robinson-Foulds (RF) distance and the pairwise weighted RF distances (Robinson and Foulds, 1981) using RAxML version 8.2.4 and the following parameter values: -f r -m GTRCATX. Because the weighted RF distances can only be calculated when bootstrap values are available, only the plain RF distances were calculated for pairwise comparisons involving the dendrograms obtained from cg/wgMLST analyses. Because cluster 2 only had two isolates, no phylogenetic tree was constructed for this cluster.

### Assessment on the Effect of Using Different Pipelines on hqSNP and Allelic Differences Identified

Per cluster, the number of SNP and allelic differences obtained from the pairwise comparisons of the isolates was compared between pipelines and between the use of a closed or draft reference in case of hqSNP analysis. Instead of comparing the absolute SNP/allelic differences between each pair of isolates, a fixed threshold was used to investigate whether the different pipelines provided concordant or discordant conclusions. Specifically, a fixed threshold of 20 hqSNP or allelic differences was used to classify two isolates as either “closely related” (less than 21 SNP or allelic differences based on wgMLST scheme) or as “loosely related” (more than 20 SNP or allelic differences). We chose to apply the same value of 20 as a fixed threshold for allelic difference as Katz et al. (2017) have previously shown a high correlation between hqSNP and wgMLST derived differences when the values were lower than 255 for *L. monocytogenes*. If one pipeline indicated that two isolates were less than 21 SNPs apart while another pipeline indicated that the same two isolates were more than 20 SNPs apart, then these two pipelines were considered discordant for these two isolates. Otherwise, the pipelines were considered concordant. The threshold used in this study is not a definitive rule but has been described by the FDA as a threshold that provides strong support for a match between two or more genomes based on hqSNP results, given that phylogenetic analyses show a monophyletic relationship of the isolates with a bootstrap support of more than 90% (Pightling et al., 2018), with classification as a “match” triggering further investigations (Wang et al., 2018).

## Results

### Quality Metrics From WGS and *de novo* Assembly

All raw sequence read files passed the post-trimming quality check with FASTQC and were then used to generate high-quality draft genomes for each of the 42 isolates used in this study. Final high-quality draft genomes ranged from 2,940,398 nt to 3,148,192 nt with a median of 3,108,633 nt (Supplementary Table S1). The N50 values ranged from 46,200 nt to 586,317 nt with a median of 161,443 nt. The number of contigs ranged from 23 to 361 with a median of 63 contigs. The average coverage ranged from 87.4× to 230.3× with a median coverage of 142.5×. Although the range of average coverage was wide, the minimum coverage obtained, 87.4×, is well above the recommended minimum average coverage to perform the analyses described here (Davis et al., 2015; Moura et al., 2016; Katz et al., 2017). No impact of different average coverages was observed in the analyses described here.

### Assessment of the Overall Relationship of the 42 *L. monocytogenes* Isolates

*In silico* 7-gene MLST-based sequence typing identified four STs, namely ST121, ST199, ST321, and ST371.

**FIGURE 1:**
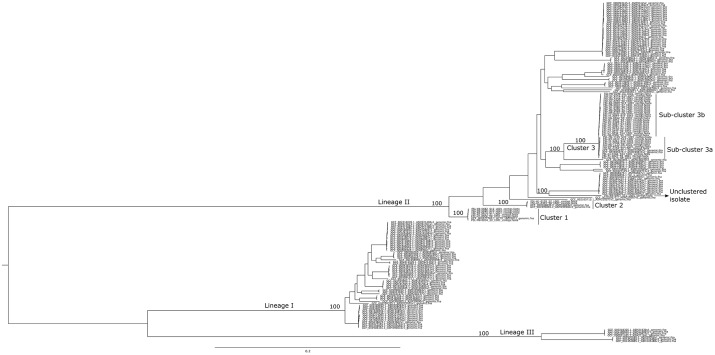
Maximum parsimony tree based on k-mer-based SNP analysis. The tree was built using kSNP3 with the core SNPs identified among the set of 42 isolates in the study dataset plus 140 *L. monocytogenes* closed genomes downloaded from the NCBI RefSeq database. Lineages (I, II, and III), the three clusters (1, 2, and 3) and sub-clusters (3a and 3b), as well as the unclustered isolate are annotated. Percentages of consensus clustering agreement across up to 100 equally parsimonious trees are shown for the clusters identified in this study and main nodes representing the *L. monocytogenes* lineages. The tree was midpoint rooted.

The 42 high-quality draft genomes were analyzed using a k-mer based approach to assess their general phylogenetic relationship and to identify suitable closed genomes to be used as references for the hqSNP pipelines. According to the maximum parsimony phylogenetic tree (Figure 1) and the *in silico* classification of the isolates based on MLST sequence typing, the 42 isolates were differentiated into three different clusters with one isolate, FSL R9-4003 [ST199], not clustering with any other isolate from the cold-smoked salmon facility dataset. The three clusters were named clusters 1 [ST121], 2 [ST371], and 3 [ST321] (Figure 1) with 6 isolates in cluster 1, 2 isolates in cluster 2, and 33 isolates in cluster 3. For all three clusters, at least one *L. monocytogenes* closed assembly from the NCBI RefSeq database clustered within the salmon facility isolates. Among the two isolates from a different cold-smoked salmon facility, FSL N1-0013 was part of cluster 1 and FSL N1-0051 was part of cluster 3.

### Comparison of CFSAN, Lyve-SET, andBN hqSNP-Based Pipelines

The hqSNP analyses using the CFSAN, Lyve-SET and BN hqSNP pipelines were run using as reference either (i) a closed genome downloaded from NCBI RefSeq database, or (ii) a high-quality draft genome from our own dataset (Table 2). This approach allowed us to compare the impact of using a publicly available completed genome (“closed reference”) versus using a less completed, high-quality draft genome from one of the isolates in the dataset, which tends to be more closely related to the isolates within each cluster (“draft reference”). A distinct “closed reference” genome was used for each of the clusters identified (i.e., clusters 1, 2, and 3); on the other hand, four “draft reference” genomes were used for separate hqSNP calling for clusters 1, 2, 3a, and 3b. This approach was needed as there was no closed genome that clustered with sub-cluster 3b.

For cluster 1, five out of the six isolates had more than 98.2% of the raw reads mapped to the closed reference genome; the sixth isolate (FSL N1-0013) had only 95.1% of the reads mapped to the closed reference genome. Five out of the six isolates had more than 99.1% of the reads mapped against the draft reference; FSL M6-0204 had 97.7% of the reads mapped to the draft reference. For the two cluster 2 isolates, approximately 94–95% of the raw reads mapped to the closed reference while more than 99.4% of the reads from both isolates mapped against the draft reference. For cluster 3, the percentage of reads that mapped against the closed reference ranged from 92.0 to 98.1%. For sub-cluster 3a, nine out of ten isolates had more than 99.1% of the reads mapped against the draft reference FSL T1-0027; FSL H6-0175 had 98.7% of the reads mapped against this reference. For sub-cluster 3b (23 isolates), more than 99.2% of the reads mapped to the draft reference. All mapping data are based on mapping done with Bowtie2 within the CFSAN pipeline. With this tool, the percentage of mapped reads was superior when the “draft reference” was used in comparison to when the “closed reference” was used.

Within cluster 1, the pairwise differences observed ranged from 1 SNP (between FSL R6-0665 and FSL R6-0682; BN pipeline with both closed and draft references) to 179 SNPs (between FSL N1-0013 and FSL M6-0204; Lyve-SET pipeline with the draft reference; Table 3 and Supplementary Table S2). The maximum pairwise difference ranged from 148 SNPs (Lyve-SET pipeline with the closed reference) to 179 SNPs (Lyve-SET pipeline with the draft reference). The mean number of SNPs found between each pair of isolates within cluster 1 was lowest when the Lyve-SET pipeline was used with a closed reference (i.e., mean pairwise difference = 79.9 SNPs) and was greatest when the Lyve-SET pipeline was used with the draft reference (i.e., mean pairwise difference = 88.3 SNPs). Hence, the Lyve-SET pipeline showed the greatest difference due to the reference used.

**Table 3:** Summary of pairwise hqSNP and allelic differences observed with different methods.

	Range of pairwise hqSNP differences (pipeline/reference)						Range of allelic differences (WGS MLST scheme)	
Cluster or sub-cluster	CFSAN		Lyve-SET		BN		cgMLST	wgMLST
	Closed	Draft	Closed	Draft	Closed	Draft		
1	2–163	2–157	2–148	2–179	1–150	1–151	0–70	0–134
2	4	4	5	5	5	5	4	4
3a	ND	1–37	ND	1–30	ND	1–30	2–20	2–46
3b	ND	0–28	ND	0–29	ND	0–27	0–15	0–32

*ND, not done. For sub-clusters 3a and 3b, only “draft references” were used because no closely related closed genome could be found to be used as a reference for sub-cluster 3b.*

For cluster 2, both Lyve-SET and BN pipelines identified five SNP differences between FSL H1-0159 and FSL H1-0322, regardless of the reference used (Table 3 and Supplementary Table S2). However, the CFSAN pipeline identified four SNP differences between these isolates when either the closed or draft references were used.

A maximum likelihood phylogenetic tree (Supplementary Figure S1) showed that cluster 3 could be further split into two sub-clusters, named sub-cluster 3a (*n* = 10) and sub-cluster 3b (*n* = 23). Both sub-clusters presented 100% bootstrap support. Since no closed genome grouped within sub-cluster 3b isolates, no further attempt to map the individual sub-cluster isolates against other closed genomes was carried out. Hence, analysis for each sub-cluster and the comparison across different pipelines was carried out solely using draft genomes from an isolate within each sub-cluster as reference. For sub-cluster 3a, the pairwise differences determined by the three pipelines ranged from 1 SNP (all pipelines) to 37 SNPs (CFSAN pipeline) with the Lyve-SET and BN pipelines yielding a maximum of 30 and 31 SNPs, respectively (Table 3 and Supplementary Table S2). Both the mean (21.3 SNPs) and median (25 SNPs) pairwise differences were greater when the CFSAN pipeline was used as compared to the Lyve-SET pipeline (mean = 16.6 SNPs; median = 19 SNPs) and the BN pipeline (mean = 17.2 SNPs; median = 18 SNPs).

For sub-cluster 3b, the pairwise differences determined by the three pipelines ranged from 0 SNP (all pipelines) to 29 SNPs (Lyve-SET) (Table 3 and Supplementary Table S2). Both the mean and median pairwise differences were very similar across the three pipelines.

### Sequence Typing, cg/wg MLST Analyses

The cgMLST and wgMLST analyses of the 42 isolates resulted in the same clustering obtained with the k-mer based SNP analysis. Generally, the range of allelic differences observed for all clusters with wgMLST was similar to the range of SNP differences obtained with the hqSNP pipelines. The allelic differences from the cgMLST analysis were typically about half the number of allelic differences identified by wgMLST, except for cluster 2, which only included two isolates that differed by only four alleles with both the wgMLST (out of 2,851 to 2,881 loci detected) and cgMLST (out of 1,678 to 1,696 loci detected) schemes (Table 3).

For cluster 1, allelic differences ranged from 0 to 134 for wgMLST (out of 2,689 to 2,812 wgMLST loci detected) and from 0 to 70 for cgMLST (out of 1,626 to 1,643 loci detected). The 10 isolates in sub-cluster 3a differed from each other by 2–46 alleles for wgMLST (out of 2,760 to 2,831 loci detected) and 2–20 alleles for cgMLST (out of 1,661 to 1,696 loci detected). The cgMLST and wgMLST results were more congruent to each other for pairs of isolates with lower number of allelic differences (i.e., more closely related isolates) as compared to pairs with higher number of allelic differences (i.e., more distantly related isolates). For sub-cluster 3b, allelic differences ranged between 0–32 and 0–15 for wgMLST (out of 2,855 to 2,940 loci detected) and cgMLST (out of 1,661 to 1,696 loci detected), respectively.

### Comparison of Tree Topologies Across Different hqSNP Pipelines

RAxML was used to compare the topologies of the maximum likelihood trees based on the hqSNP matrices obtained from each pipeline/reference combination for cluster 1 and each pipeline using the draft reference for sub-clusters 3a and 3b. In order to objectively compare the tree topologies across the different methods, the pairwise RF distances and the pairwise weighted RF distances between each pair of trees was computed. While the weighted RF distance takes into account the number of different splits (bipartition) and the bootstrap support for each different split between two trees, the RF distance takes into account only the number of different splits between two trees. RF distances range from 0 (when the trees are identical) to 2^∗^(*n* - 3), where *n* is the number of taxa in the tree. Hence, if two trees with the same five taxa differ by 1 split, their RF distance is 1 out of 4 (i.e., 25% of the splits are different). Since cluster 2 only had two isolates, no phylogenetic trees were constructed for this cluster.

For cluster 1, isolates FSL R6-0665, FSL R6-0670, and FSL R6-0682 clustered together in the maximum likelihood phylogenetic trees with 100% bootstrap support (Supplementary Figure S1). The tree generated from the Lyve-SET pipeline using the “closed reference” genome was the only tree to differ from all the others with an RF = 2 and a weighted RF = 1.0 (Table 4). However, the difference between the trees was solely due to the clustering of isolates FSL M6-0204, FSL N1-0013, and FSL H1-0506. Since these isolates showed an average SNP difference greater than 50 SNPs, this difference in the tree topology would be unlikely to affect the conclusions of a source tracking investigation.

**Table 4:** Pairwise unweighted and weighted Robinson-Foulds (RF) distances for different methods.

Method 1 (pipeline/reference)	Method 2 (pipeline/reference type)	Unweighted RF distance	Weighted RF distance
*Cluster 1* (maximum possible RF value = 6)			
Lyve-SET/draft	Lyve-SET/closed	2	1.00
Lyve-SET/draft	CFSAN/closed	0	0.00
Lyve-SET/draft	CFSAN/draft	0	0.00
Lyve-SET/draft	BN/closed	0	0.00
Lyve-SET/draft	BN/draft	0	0.00
Lyve-SET/draft	cgMLST/NA	4	ND
Lye-SET/draft	wgMLST/NA	4	ND
Lyve-SET/closed	CFSAN/closed	2	1.00
Lyve-SET/closed	CFSAN/draft	2	1.00
Lyve-SET/closed	BN/closed	2	1.00
Lyve-SET/closed	BN/draft	2	1.00
Lyve-SET/closed	cgMLST/NA	2	ND
Lyve-SET/closed	cgMLST/NA	2	ND
CFSAN/draft	BN/closed	0	0.00
CFSAN/draft	BN/draft	0	0.00
CFSAN/draft	cgMLST/NA	4	ND
CFSAN/draft	wgMLST/NA	4	ND
CFSAN/closed	CFSAN/draft	0	0.00
CFSAN/closed	BN/closed	0	0.00
CFSAN/closed	BN/draft	0	0.00
CFSAN/closed	cgMLST/NA	4	ND
CFSAN/closed	wgMLST/NA	4	ND
BN/draft	cgMLST/NA	4	ND
BN/draft	wgMLST/NA	4	ND
BN/closed	BN/draft	0	0.00
BN/closed	cgMLST/NA	4	ND
BN/closed	wgMLST/NA	4	ND
wgMLST/NA	cgMLST/NA	0	ND
*Sub-cluster 3a* (maximum possible RF value = 14)			
Lyve-SET/draft	CFSAN/draft	2	0.72
Lyve-SET/draft	BN/draft	2	0.72
Lyve-SET/draft	cgMLST/NA	2	ND
Lyve-SET/draft	wgMLST/NA	6	ND
CFSAN/draft	BN/draft	0	0.00
CFSAN/draft	cgMLST/NA	2	ND
CFSAN/draft	wgMLST/NA	4	ND
BN/draft	cgMLST/NA	2	ND
BN/draft	wgMLST/NA	4	ND
cgMLST/NA	wgMLST/NA	6	ND
*Sub-cluster 3b* (maximum possible RF value = 40)			
Lyve-SET/draft	CFSAN/draft	18	4.51
Lyve-SET/draft	BN/draft	16	3.80
Lyve-SET/draft	cgMLST/NA	24	ND
Lyve-SET/draft	wgMLST/NA	18	ND
CFSAN/draft	BN/draft	14	1.62
CFSAN/draft	cgMLST/NA	22	ND
CFSAN/draft	wgMLST/NA	20	ND
BN/draft	cgMLST/NA	22	ND
BN/draft	wgMLST/NA	18	ND
cgMLST/NA	wgMLST/NA	22	ND

*ND, not defined. For cgMLST and wgMLST, no reference genome is used; the weighted RF distance cannot be calculated because no bootstrap value is obtained from MLST analyses.*

For sub-cluster 3a, the tree topologies obtained with the CFSAN and BN pipelines were identical. Once again, the Lyve-SET pipeline resulted in a tree topology that differed from the other two pipelines. The difference between the trees involved three pairs of isolates, FSL T1-0261 and FSL T1-0938, FSL M6-1145 and FSL R9-4443, and FSL R9-4438 and FSL M6-1133. These three pairs were consistently grouped together; however, in the Lyve-SET tree, the positions of the pairs were in a different order when compared with the CFSAN and BN trees (Supplementary Figure S1). The RF distance between the Lyve-SET tree and the other two trees was 2 but the weighted RF was only 0.72 (Table 4) because the position of the pairs had low bootstrap support (bootstrap = 0.42 in the Lyve-SET tree and 0.30 in the CFSAN and BN trees).

For sub-cluster 3b, the largest cluster identified, all three trees differed (Table 4); for this cluster, analyses were only performed with the “draft reference” genome. The two most similar trees were the ones obtained with the CFSAN pipeline and the BN pipeline (RF = 14, weighted RF = 1.62). The most dissimilar trees were the ones obtained with the Lyve-SET pipeline and the CFSAN pipeline (RF = 18, weighted RF = 4.51). All branches with bootstrap support greater than 90% were consistent across the three trees suggesting that if bootstrap support is taken into consideration, these differences would be unlikely to affect the conclusions of a source tracking investigation.

### Comparison of Dendrogram Topologies From cg/wgMLST Pipelines Against Phylogenetic Trees From hqSNP Pipelines

The same approach described above was used to compare the dendrograms generated from the cgMLST and wgMLST pipelines between each other and to the phylogenies generated by each of the hqSNP pipelines. Since the dendrograms were constructed based on similarity matrices, no bootstrap values were calculated to assess the confidence of each node. Therefore, only the plain RF distances were obtained for these comparisons. In general, the cgMLST and wgMLST dendrograms showed a high RF distance; similarly, comparisons between cg/wgMLST dendrograms and hqSNP phylogenies generally showed high RF distances (Table 4). For cluster 1, no difference was observed between the cgMLST and the wgMLST dendrograms. However, the dendrograms differed from the phylogenetic trees with RF distances ranging from 2 to 4. For sub-cluster 3a, the pairwise comparison of the cgMLST and wgMLST dendrograms had an RF distance of 6, which was the same distance obtained when the wgMLST dendrogram was compared with the Lyve-SET/draft genome phylogenetic tree. For sub-cluster 3a, the RF distance between the cgMLST dendrograms and the phylogenetic trees was smaller compared to the RF distance between the wgMLST dendrogram and phylogenetic trees. For sub-cluster 3b, the cgMLST and wgMLST dendrograms showed an RF distance of 22. The dendrograms showed RF distances ranging from 18 to 24 when compared against the phylogenetic trees. In contrast to the observations of sub-cluster 3a, the sub-cluster 3b RF distances between the cgMLST dendrogram and phylogenetic trees were generally higher compared to the RF distances of wgMLST dendrogram and the phylogenetic trees.

### Impact on Result Inference by Comparing Pairwise Differences Across Pipelines

A fixed threshold of 20 hqSNP/wgMLST allelic differences was used to classify the pair of isolates into “closely related” and “loosely related” within each cluster and sub-cluster. When two methods were compared, results for each isolate pair were considered concordant if both methods classified the same pair as “closely related” or “loosely related.” If one method classified the pair as “closely related” and the other method classified the same pair as “loosely related,” then the results were considered discordant.

For all three hqSNP pipelines, changing the reference sequence from one of an isolate within our dataset (“draft reference”) to the closest related closed genome downloaded from NCBI (“closed reference”) did not result in discordant results for cluster 1 (Table 5) and cluster 2 (data not shown). For sub-cluster 3a, 64% and 24% discordant results were observed for the CFSAN and Lyve-SET pipelines, respectively, when a different reference genome was used, likely due to the fact that a number of isolate pairs in this sub-cluster differed by SNP differences around 20, so that a small change in SNP numbers would change classification. For sub-cluster 3b, 19% and 12% discordant results were found with the CFSAN and Lyve-SET pipelines, respectively, when the reference was different. The BN hqSNP pipeline provided 100% concordant results for cluster 1 and sub-clusters 3a and 3b when either closed or draft references were used. Consistent with this, the BN hqSNP pipeline also showed the highest proportion of pairwise comparisons with identical SNP values when changing the reference.

**Table 5:** Impact on results inference by changing reference in hqSNP analysis.

		Concordant results		
Cluster	Pipeline	Equal SNP values (%)	Different SNP values (%)	Discordant results (%)
1	CFSAN	27	73	0
	Lyve-SET	20	80	0
	BN	67	33	0
3a	CFSAN	2	33	64
	Lyve-SET	9	67	24
	BN	80	20	0
3b	CFSAN	1	80	19
	Lyve-SET	23	65	12
	BN	91	9	0

*A theoretical SNP threshold of 20 SNPs applied to assess the impact on results inference.*

The same 20 SNP threshold was applied to determine the impact of changing pipelines while using the same reference (Table 6). wgMLST allelic differences were also included in this analysis. All three hqSNP pipelines and wgMLST approach provided concordant results for cluster 1 when either the draft or closed references were used. For sub-cluster 3a, the highest percentage of discordant results was observed when the CFSAN hqSNP pipeline was compared against the Lyve-SET or BN hqSNP pipelines. For sub-cluster 3b, the highest percentage of discordant results was observed from the wgMLST analysis compared to the hqSNP pipelines. Amongst the hqSNP pipelines, the most discordant results were observed when the BN hqSNP pipeline was compared against the CFSAN or Lyve-SET pipelines. Hence, the proportion of discordant results depended on the cluster that was analyzed, the reference type and the pipeline used.

**Table 6:** Discordant conclusions (%) obtained due to changing approaches for pairwise comparison between strains.

Cluster	Reference genome	Pipelines	Pipelines			
			CFSAN	Lyve-SET	BN	wgMLST
1	Draft	CFSAN	–	0	0	0
		Lyve-SET	0	–	0	0
		BN	0	0	–	0
		wgMLST	0	0	0	–
1	Closed	CFSAN	–	0	0	0
		Lyve-SET	0	–	0	0
		BN	0	0	–	0
		wgMLST	0	0	0	–
3a	Draft	CFSAN	–	36	33	13
		Lyve-SET	36	–	11	18
		BN	33	11	–	18
		wgMLST	13	18	18	–
3b	Draft	CFSAN	–	7	14	16
		Lyve-SET	7	–	9	17
		BN	14	9	–	24
		wgMLST	16	17	24	–

### Assessment of Environmental Contamination Patterns and Persistence

**FIGURE 2:**
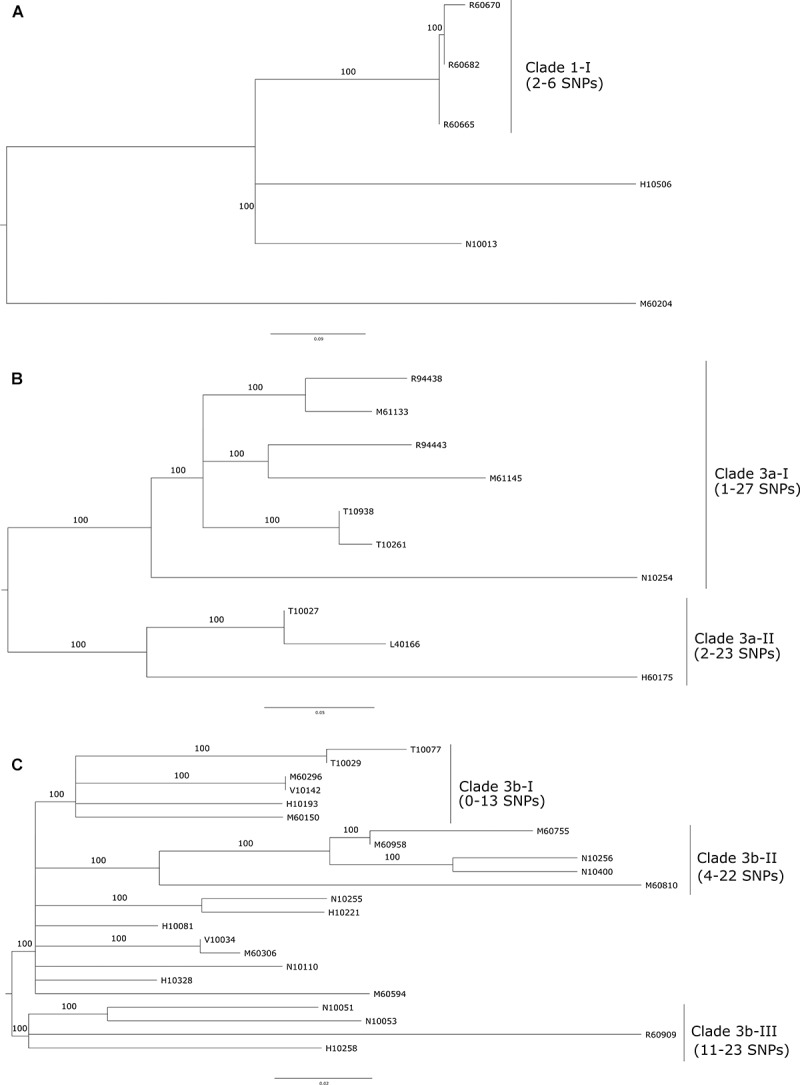
Maximum likelihood phylogenetic tree based on hqSNP analysis using the BioNumerics (BN) pipeline. The tree was constructed with RAxML using core hqSNPs identified within cluster 1 **(A)**, sub-cluster 3a **(B)**, and sub-cluster 3b **(C)**. Bootstrap values greater than 70% are shown above the branches. Clades within (sub-) clusters are shown with the hqSNP ranges identified with the three hqSNP methods (CFSAN, Lyve-SET, and BN).

For cluster 1 (Figure 2A), three of the isolates (i.e., FSL R6-0665, FSL R6-0682, and FSL R6-0670) clustered together with 100% bootstrap value (clade 1-I). These isolates were all collected in September 2007 and few SNPs/allelic differences were observed among them (≤6 SNPs), suggesting a common source. Two other cluster 1 isolates (FSL H1-0506 and FSL M6-0204) were obtained in 2000 and 2011, respectively; these isolates differed by more than 63 SNPs by all hqSNP pipelines (more than 63 alleles by wgMLST) from each other as well as the three 2007 isolates, suggesting that a common contamination source is unlikely. It is also unlikely that the cluster 1 isolates characterized here represent a persistent strain. FSL N1-0013, the final isolate in cluster 1 was isolated from a different processing plant in 1998; this isolate differed by more than 63 SNP (more than 45/alleles) from all other isolates in this cluster. The two cluster 2 isolates, collected in March and July 2000 from the same site (“drain 54”), showed few SNP/allele differences (pairwise difference = 4–5 SNPs/alleles), suggesting a common source and/or persistence over at least a few months.

Sub-cluster 3a (Figure 2B) includes one monophyletic clade (3a-I; 100% bootstrap support) with seven isolates that shows a maximum of 27 SNP differences; these seven isolates were obtained between 1998 and 2015, suggesting the possibility of persistence for more than 16 years (or multiple re-introduction events over more than 16 years). Within 3a-I, there are three pairs of closely related isolates, including (i) FSL M6-1145, collected in 2012, and FSL R9-4443, collected in 2015 (less than 10 SNPs across all methods used); (ii) FSL M6-1133 (collected in 2012) and FSL R9-4438 (collected in 2015) (less than 7 SNPs across all the methods used); and (iii) isolates FSL T1-0261 and FSL T1-0938, which were collected in close temporal proximity (July and November 2001) and showed one SNP difference. Another isolate, FSL N1-0254 (collected in October 1998) showed a range of 17–24 and 18–25 SNPs, depending on the hqSNP method, in comparison to isolates FSL T1-0938 and FSL T1-0261, respectively. Sub-cluster 3a included one additional clade of three closely related isolates (clade 3a-II; 100% bootstrap support); FSL T1-0027, collected in March 2001 and FSL L4-0116, collected in October 2002, showed two to three SNPs differences between each other, and FSL H6-0175, collected in June 2004, showed 14–20 and 16–23 SNP differences, depending on the hqSNP method used, against FSL T1-0027 and FSL L4-0116, respectively. While the maximum SNP difference within sub-cluster 3a (clades 3a-I and 3a-II combined) ranged from 30 to 37 SNPs, depending on the hqSNP method used, the minimum SNP difference between a clade 3a-I and a clade 3a-II isolate ranged from 17 to 25 SNPs, depending on the hqSNP method used. This range of SNP differences suggests that sub-cluster 3a represents a single persistent strain, which may be in the process of diversification into two distinct strains represented by clades 3a-I and 3a-II.

For the 23 isolates in sub-cluster 3b (Figure 2C), the range of SNP differences was 0–29 (Lyve-SET pipeline); the isolate dates ranged from August 1998 to February 2012. All isolates within sub-cluster 3b showed ≤20 SNP differences to at least one other isolate within this sub-cluster. The SNP difference range and monophyletic clustering of sub-cluster 3b could be interpreted as suggesting that these isolates represent a common source and possible a population that persisted in this facility over at least 14 years. Within sub-cluster 3b there are three well supported (greater than 95% bootstrap support) clades of more closely related isolates, including (i) 3b-I (six isolates with isolation dates of March 2001 to June 2011, 0–13 SNP differences), (ii) 3b-II (five isolates with isolation dates of October 1998 to February 2012, 4–22 SNP differences), and (iii) 3b-III (four isolates with isolation dates of August 1998 to December 2007, 11–23 SNPs). Interestingly, clade 3b-II represents five isolates from drains, including three isolates obtained from the “sturgeon room drain”; similarly, clade 3b-I includes two isolates, FSL V1-0142 (collected in October 2009) and FSL M6-0296 (collected in June 2011), which were both from drains and showed 0 SNP differences. Two other isolate pairs, which were not part of clades 3b-I, 3b-II, or 3b-III, showed less than eight SNP differences: (i) FSL V1-0034 (collected in February 2009) and FSL M6-0306 (collected in June 2011), and (ii) FSL N1-0255 (collected in October 1998) and FSL H1-0221 (collected in February 2000). Isolate FSL N1-0051 (collected in 1998) originated from a different facility but is part of clade 3b-III and shows 11–23 SNP differences to other isolates in this clade, indicating a possible common source of this isolate and isolates obtained from the main facility studied here.

The WGS results and the accompanying metadata of the isolates suggest that at least three strains persisted in the facility for more than 3 months. Cluster 2 (two isolates) represents a strain that persisted for at least 4 months in 2000, but this strain was not found in other years; sub-cluster 3a represents a strain (10 isolates) that persisted for at least 17 years from 1998 to 2015; sub-cluster 3b (23 isolates) represents a strain that persisted for at least 14 years from 1998 to 2012. Though it is most likely that isolates could have persisted and diversified within this facility, multiple re-introduction of some of the isolates from an external source cannot be fully excluded either.

## Discussion

Since the last decade, WGS has been increasingly used as a subtyping method for outbreak investigations involving foodborne pathogens including *L. monocytogenes* (Schjorring et al., 2017; Maurella et al., 2018), *Salmonella* (Donachie et al., 2018; Marshall et al., 2018) and Shiga-toxin producing *Escherichia coli* (Mylius et al., 2018). Among the different WGS data analysis approaches developed, Lyve-SET, the hqSNP pipeline developed by the United States CDC (Katz et al., 2017) and the CFSAN hqSNP pipeline, developed by the FDA (Davis et al., 2015) as well as their dependencies (programs) are publicly available and can be acquired with no cost. Another approach to WGS-based subtyping relies on allele differences within a subset of genes instead of hqSNP differences. The cgMLST scheme is freely available and can be run locally (requiring bioinformatics competences) or using the online BIGSdb-Lm platform (no bioinformatics expertise required). End-users with limited bioinformatics expertise can also use proprietary softwares to carry out cg/wgMLST analysis. While different pipelines have been evaluated for clinical isolates, the food industry requires guidance for pipelines to be used for comparisons among food and food associated isolates. We thus used three hqSNP pipelines (Lyve-SET, CFSAN, and BN) and two WGS-based MLST schemes (wgMLST and cgMLST) to analyze a longitudinal dataset consisting of 40 *L. monocytogenes* isolates obtained from a cold-smoked salmon facility between 1998 and 2015 and previously characterized as a single subtype (ribotype DUP-1062), suggesting possible persistence.

### K-Mer Based SNP Analysis and MLST Based Approaches Are Likely to Yield Comparable Clustering of *L. monocytogenes* Isolates

K-mer based subtyping methods are considered less discriminatory and produce less epidemiologically concordant results as compared to either hqSNP and cg/wgMLST methods and, therefore, are mainly used for a crude clustering of isolates and assessment of their relatedness (Nadon et al., 2017). The k-mer based SNP analysis indicated that the 40 isolates representing the longitudinal isolate set from a single smoked seafood processing facility could be differentiated in three different clusters and one unclustered isolate. Both wgMLST and cgMLST analysis resulted in the same clustering as the k-mer based method indicating that both these approaches could be used for the initial assessment of the relatedness of an isolate set. K-mer based methods have the advantage of not requiring a curated database or a reference genome for analysis, which are pre-requisites for the cg/wgMLST approaches and hqSNP pipelines, respectively (Nadon et al., 2017). Though k-mer analysis were used here to identify the reference sequence genomes for hqSNP analysis, our data shows that cg/wgMLST approaches could have also been used for this purpose. While our data thus indicate some value of initial k-mer based WGS data analyses, the concordant clustering obtained with k-mer and cg/wgMLST-based methods suggests that cg/wgMLST data can be used for initial clustering and to identify reference genomes for hqSNP data analyses.

### cgMLST Provides Lower Numbers of Allelic Differences, but Similar Clustering and Relatedness Assessments as Compared to hqSNP and wgMLST

cgMLST clustering correlated well with the k-mer based, hqSNP and wgMLST clustering as well as traditional 7-gene MLST groupings, despite the fact that 7-gene MLST is clearly less discriminatory than WGS-based methods. The reduced discriminatory ability of 7-gene MLST is supported by the fact that isolates in sub-clusters 3a and 3b all represented the same 7-gene MLST ST, while clearly being differentiated by cgMLST. Classification of isolates into 7-gene MLST STs [and associated clonal complexes (CCs)], however, may still be valuable as large historical data based on 7-gene MLST STs exists, which may allow for isolate comparisons that identify specific phenotypes typically associated with a given ST or that identify STs or CCs that have been associated with specific outbreaks (Maury et al., 2016). For example, the cluster 1 isolates from this study here were classified into ST121, which has previously been reported to be common in food processing facilities, but rare in human clinical cases (Henri et al., 2016) and has been associated with presence of quaternary ammonium resistance genes (Pasquali et al., 2018). In the future, classification into 7-genes MLST STs and CCs likely will be increasingly integrated into WGS analysis pipelines, but may also become less important as larger WGS databases that include more historical isolates become available.

For the *L. monocytogenes* isolates characterized here, allelic differences obtained by cgMLST were usually lower in comparison to wgMLST or hqSNP differences. This can be easily explained by the fact that both wgMLST and hqSNP analyses consider a much higher percentage of the genome when compared to cgMLST. In addition, the cgMLST scheme is composed mostly by slowly evolving genes. Using *L. monocytogenes* genomes available at the time, the evolution rate of cgMLST types has previously been estimated to be around 0.2 alleles per year (Moura et al., 2016), suggesting that differentiation of isolates that share a recent common ancestor within 5 years may not always be possible. Therefore, from a source tracking investigation standpoint, the use of more discriminatory wgMLST or the hqSNP analysis approaches often is considered essential, particularly when a dataset includes isolates that are highly likely to be closely related to each other. Despite these, largely theoretical, considerations, cgMLST provided for appropriate initial assessment of isolate relatedness for the data set analyzed here. This may reflect that isolates from food processing plants and food associated environments that represent a suitable environment for *L. monocytogenes* growth (such as the source facility for the isolates characterized here) may allow for diversification, but also may indicate that tracking *L. monocytogenes* sources often assesses persistence for long time intervals (more than 4 years) (Ferreira et al., 2014). Overall, based on this data set, cgMLST based analysis provided the information needed for *L. monocytogenes* source tracking, including persistence assessment. Previous studies using the same cgMLST scheme used here (Moura et al., 2016, 2017; Chen et al., 2017) or different cgMLST schemes (Chen et al., 2016) also showed that cgMLST is, in most cases, sufficient to identify clonal groups and discriminate outbreak strains from epidemiologically unrelated strains of *L. monocytogenes*. However, subsequent wgMLST and/or hqSNP analysis may still be desired in investigations of persistence or in source tracking investigations where accurate assessment of divergence dates is essential. Importantly, the cgMLST scheme is publicly available from http://bigsdb.pasteur.fr/listeria/ allowing industry and others to perform cgMLST without the need to invest in commercial software. Proprietary software like BioNumerics makes the analysis accessible to users with limited bioinformatics expertise.

### Both wgMLST or hqSNP Represent Suitable Approaches for WGS-Based Data Analyses With Higher Resolution Than cgMLST

Despite the fact that cgMLST provided what appeared to be appropriate discrimination among the isolates characterized here, our data also confirmed the increased discriminatory power of wgMLST or hqSNP approaches. Industry may hence also elect to perform further in-depth data analyses in addition to cgMLST, using wgMLST or hqSNP approaches where accurate assessment of divergence dates is essential. For instance, this is highly relevant when making decisions on the origin of contamination particularly for isolates sampled within a short timeframe. Importantly, we showed that similar discriminatory power was achieved for both wgMLST and hqSNP; specifically, the range of allelic differences obtained with wgMLST is comparable to the range of hqSNP differences obtained with the hqSNP pipelines across all clusters investigated. This is also consistent with previous studies that have shown similar discriminatory power for wgMLST or hqSNP approaches for *L. monocytogenes*, based on isolate sets that largely represented human clinical isolates (Chen et al., 2017; Katz et al., 2017); Katz et al. (2017) specifically showed that the Lyve-SET hqSNP pipeline and wgMLST approach are highly concordant for *L. monocytogenes* isolates with less than 255 hqSNP differences (Katz et al., 2017). Overall, this suggests that either hqSNP or wgMLST approaches would typically be appropriate for detailed analyses of *L. monocytogenes* isolate sets that are identified as closely related by initial cgMLST analyses.

Importantly, in addition to WGS-derived measures of isolate similarity (e.g., number of SNP differences), “trees” derived from WGS data (e.g., phylogenetic trees for hqSNP data, dendrogram for MLST data) can provide important information when assessing the relationship between isolates (Chen et al., 2017; Pightling et al., 2018). This is supported by Pightling et al. (2018), who suggested that a fixed SNP threshold (e.g., 20 SNPs for *L. monocytogenes*) can be used as a conservative threshold that provides support for a “match” between two or more genomes, given that phylogenetic analyses show a monophyletic relationship with a bootstrap support greater than 90% (Pightling et al., 2018).

Hence, constructing appropriate trees with confidence measures (such as bootstrap values) can be important not only for outbreak investigations, but also when using WGS data for source tracking. With regard to the specific phylogenies constructed for cluster 1 as well as sub-clusters 3a and 3b, our data indicate that all three hqSNP pipelines resulted in the same conclusions regarding which isolates were closely related if both hqSNP differences and the phylogenetic tree topology, including the split bootstrap support, were taken into account. This suggests that any of the three hqSNP pipelines evaluated here could be used to construct phylogenetic trees that can be used to clarify the relationships between closely related *L. monocytogenes* isolates. In addition, the wgMLST-based dendrograms generally identified the same clades of closely related isolates with a common recent ancestor as did the hqSNP phylogenetic trees. However, wgMLST trees often differed from hqSNP phylogenies with regard to the placement of isolates where clustering in the hqSNP trees were not strongly supported by greater than 90% bootstrap values. This supports the importance of having trees with some measure of confidence for a given node, which unfortunately is often not available for wgMLST or cgMLST based dendrograms that are based on allelic type similarity matrices. It is, however, possible to construct cg/wgMLST-based phylogenies with bootstrap support, particularly when concatenated sequences rather than allelic profiles are used as inputs, even though these approaches may not always be easily accessible to individuals with limited bioinformatics expertise. Hence, use of hqSNP-based approaches for analysis of WGS data, subsequent to initial cgMLST analyses, may be preferable if access to phylogenies with bootstrap support for nodes is desired. Importantly, our findings also indicated that commercial software packages with a graphical interface can be used both for cg/wgMLST and hqSNP analysis and hence would allow for both primary cgMLST analyses followed by secondary wgMLST or hqSNP based analyses, including construction of hqSNP-based trees with bootstrap support. Availability of such alternatives to Linux-based bioinformatics approaches lowers the threshold to access bioinformatics for the food industry microbiologists and can serve as an easy to use “entry level” analytical tool.

### While Use of Closely Related Reference Genomes Is Essential for Reliable hqSNP Calling, Choice of Closed or Draft Genomes Has Limited Impacts on SNP Difference Counts, Clustering, or Other Conclusions Based on WGS Data

Unlike for cgMLST or wgMLST, which do not require selection of reference genomes, using a closely related genome as reference is essential for hqSNP data analyses to prevent misalignment of the reads against the reference genome and misidentification of SNPs (Pightling et al., 2014; Kwong et al., 2016). While identification of closely related reference genomes in public databases may be time consuming and challenging, use of a genome that represents the most high-quality assembly that is obtained when a set of closely related isolates is sequenced (i.e., a “draft reference genome”) represents a possible alternative to selection of a “closed reference genome” from a public database. In our analyses, changing from a “closed reference genome” to a “draft reference genome” had limited impact on conclusions obtained with the three hqSNP pipelines used here, particularly when the isolates were closely related (≤20 SNP differences). This is consistent with previous studies that also showed that use of “closed reference genomes” for hqSNP calling in *L. monocytogenes* provided similar SNP numbers (Portmann et al., 2018) and resolution and phylogeny reconstruction as was obtained when using “draft reference genomes” for SNP calling (Kwong et al., 2016).

This is important as identifying a suitable, closely related, “closed genome” to be used as a reference may be time-consuming and require computer skills that may not be easily accessible; in some cases (as for sub-cluster 3b here) a suitable closely related reference genome may not even be available. Our observation that use of a closely related “draft reference genome,” which should be available any time more than two closely related isolates are sequenced, represents an appropriate approach to hqSNP data analyses in *L. monocytogenes* provides important information that will facilitate and simplify use of hqSNP-based data analyses approaches, including by industry.

### A Fixed Threshold Is a Useful Starting Point for WGS Based Investigation but Cannot Be Used to Dichotomize Isolate Relatedness (“More Similar or Not”); Isolates Relatedness Needs to Be Assessed on the Context of Appropriate Epidemiological Evidence and Metadata

Cut-off values are valuable to provide a starting point for an investigation, whether it may be an outbreak investigation or a root-cause analyses type investigation in a processing facility. For cgMLST, a cut-off of 7 allelic differences has been validated for epidemiologically related isolates (Moura et al., 2016). However, many publications emphasize that the use of a static hqSNP or allele number as a cut-off to classify isolates as “related” or “unrelated” is not appropriate, particularly when one tries to establish cause and effect type relationships (e.g., food X was responsible for human case Y; ingredient A was responsible for contamination of finished product B) (Jagadeesan et al., 2018). Our data show that use of a fixed threshold can be problematic when different analysis methods and approaches are used, particularly if isolates show numerical SNP or allelic differences that are close to a chosen cut-off. For example, in sub-cluster 3a, where isolates differed by a mean of 21.3 SNPs using the CFSAN hqSNP pipeline, changing the reference genome from a “close genome” to a “draft genome,” changed the classification of isolates into “closely related” (less than 20 SNP differences) or “loosely related” for 64% of the isolate pairs. These results further support that it is difficult to define an absolute hqSNP/allelic threshold as minor differences will be observed due to the choice of analysis approaches and reference genomes in hqSNP analysis. This further supports the need to have a “sliding window” approach to interpret hqSNP/allelic differences which should be considered along with supporting evidence to determine the relatedness between isolates and the overall implications of the relatedness identified, which is in agreement with the recommendations of Pightling et al. (2018). For example, an evidence could initially be evaluated when using a cut-off of 20 SNPs, followed by re-evaluation of evidence when 15, 10, and 5 SNPs are used as cut-off, with the possibility of also using larger SNP-cut-off values (e.g., 25, 30). While this approach can be easily used in outbreak investigations by simply assessing food exposure data (e.g., case control study data) when different SNP cut-offs are used to define “cases,” application of this approach in root cause analysis for contamination events is more challenging as these investigations may lack a well-defined outcome, such as “case” or “control.” However, collection and availability of better metadata for food associated isolates will facilitate future application of this type of approach.

Use of fixed thresholds to classify isolates as “microbiologically related” or “microbiologically unrelated” is also problematic as bacteria can show considerable differences in generation time depending on their environment. Possible generation times for *L. monocytogenes* could range from around 30 min (under “perfect” conditions for growth) to generation times of multiple days or considerably longer (e.g., under conditions that allow for survival, but not growth, such as freezing) with much more rapid accumulation of mutations over calendar time (e.g., years) when bacteria show consistent rapid growth. These considerations are the reason why it is often emphasized that in addition to WGS-derived measures of genetic similarities (as well as tree topologies and support for a given clade), other factors such as the ability of the organism to multiply in a facility, exposure to stress and adaptation, delineation of potential transmission routes must be considered when assessing the implications of identifying closely related isolates in different samples. The importance of these elements can be highlighted from the fact that FSL N1-0051 isolated in 1998 was part of sub-cluster 3b and showed less than 20 SNP differences with 18 out of 23 isolates in this sub-cluster, though this isolate originated from a different food facility. The occurrence of identical *L. monocytogenes* isolates in different geographical regions has been previously shown by Stasiewicz et al. (2015). While these two food facilities may share a common supplier, additional detailed information would be required to make correct interpretations, including to determine whether the specific source for isolate FSL N1-0051 may be related to other isolates in sub-cluster 3b, including a possible shared raw material supply chain, such that isolates classified into sub-cluster 3b were introduced into both facilities from that common supplier.

Similar to the need for having epidemiological evidence in an outbreak investigation to support WGS-derived genetic similarities (Chen et al., 2017; Nadon et al., 2017; Allard et al., 2018; Pightling et al., 2018), additional detailed evidence is needed to interpret WGS-derived genetic similarities when investigating microbial contamination events in a supply chain or a processing facility (Pightling et al., 2018). Hence, collection of detailed metadata for bacterial isolates collected from foods and food-associated environments is essential to allow for meaningful interpretation of WGS data collected for these isolates. The importance of detailed metadata and epidemiological information for interpretation of WGS data is also supported by the overall WGS data generated here for isolates collected from a specific processing facility over 18 years.

Based on the SNP/allele differences and clustering of isolates, we suggest that at least three strains, represented by cluster 2, sub-cluster 3a, and sub-cluster 3b, have persisted in the facility environment, each for a period greater than 3 months. Sub-cluster 3a also had two clades (3a-I and 3a-II) where isolates from one clade differed from the isolates from the other clade by at least 13 cgMLST allelic differences, which would place their most recent common ancestor about 65 years in the past, well within the age of the building (more than 100 years) (Lappi et al., 2004), with an estimated evolution rate of 0.2 cgMLST alleles per year (Moura et al., 2016). We, however, cannot rule out the possibility that sub-cluster 3a isolates evolved outside the facility and were introduced multiple times independently after their divergence into clades 3a-I and 3a-II. Within the facility there may be environments, such as post-processing cold rooms, where the low temperature and limited availability of nutrients may result in an increased generation time (i.e., cells take longer to divide), which could lower the rate of allelic and SNP diversification that was previously estimated heavily based on clinical *L. monocytogenes* genomes (Moura et al., 2016). Conversely, other environments within the facility, such as drains in a raw product production area, may allow for shorter generation times (i.e., cells divide faster), which could increase the rate of allelic and SNP diversification. Data on the exact location, and conditions found in these locations (i.e., temperature, presence of raw material, sanitation procedures, humidity, etc…), where isolates were collected, thus would be important to assess whether the numbers of SNP or allelic differences are compatible with persistence in that location. Importantly, the source facility for the 40 isolates characterized here has been included in a number of previous studies, which have characterized *Listeria* and *L. monocytogenes* contamination and persistence patterns over more than 10 years (Hoffman et al., 2003; Lappi et al., 2004; Thimothe et al., 2004; Hu et al., 2006; Malley et al., 2013; Vongkamjan et al., 2013). Previous studies have suggested persistence of a number of *L. monocytogenes* ribotypes in this facility, including ribotype pattern DUP-1062 and specifically subtype DUP-1062A (Lappi et al., 2004; Thimothe et al., 2004). Subsequent PFGE subtyping showed that isolates with ribotype 1062A represented 5 distinct PFGE profiles (Vongkamjan et al., 2013). However, unlike WGS, PFGE and ribotype data can not be used for phylogenetic analyses. While further WGS-based characterization of isolates with ribotype DUP-1062 showed the improved ability of WGS to elucidate transmission patterns, the data reported here also showed the challenges that remain with interpretation of subtype data, particularly when trying to differentiate persistence and re-introduction, which represents a particular challenge for facilities where the product does not undergo a kill step (which would eliminate pathogens introduced with raw material) and where limited metadata (including on barriers to re-introduction and details on supply chains) are available. However, large subtype datasets that details subtype frequency in an overall supply chain can be used to provide statistical support for persistence, as previously reported with ribotype data for the facility studied here and other similar facilities (Norton et al., 2001a; Malley et al., 2013). As larger WGS data sets with appropriate metadata become available, there thus will be a need to further develop and adopt modeling-based and statistical approaches that will allow for more formal analysis of WGS data to define evidence for persistence. Further efforts in associating old PFGE-based subtypes with WGS-based subtypes, such as the PFGE-MLST dictionary developed by Maury et al. (2016), would allow for data on older isolates, which have been subtyped by PFGE, to be assessed in the light of current WGS data.

## Conclusion

Overall, our data suggest that WGS data analysis approaches that can be used by the food industry for source tracking in the event of a microbial contamination are currently available, both with graphical commercial platforms if no bioinformatics competence is available as well as with more flexible and configurable free software when genomics and bioinformatics competences are available. A relatively simple workflow includes initial cgMLST analyses that can be followed by wgMLST and/or hqSNP data analysis for increased resolution. While hqSNP-based phylogenies can provide confidence measures for different nodes, hqSNP and wgMLST derived trees provided comparable topologies and distance measures, at least for this food facility *L. monocytogenes* case study. Importantly, however, it is likely that isolate metadata often are insufficient to allow for appropriate analysis and interpretation of WGS-based subtype data, particularly since a single static SNP or allelic difference cut-off cannot be used to classify isolates as “related” or “not related.” Hence, future efforts will need to include a focus on collection of sufficient metadata when bacterial isolates that may be used for subtyping are collected from food or through monitoring of food associated environments, including by academic research projects, which represented the sources of the isolates characterized here. The metadata must also be (i) cleaned to remove replicates, unintended information, typos, and (ii) harmonized to allow for information exchange and cross-reference between laboratories.

## Author Contributions

BJ and LB performed wgMLST, cgMLST, and hqSNP analyses using BioNumerics as well as comparative analyses across different methods. RO selected the isolates and performed the CFSAN and Lyve-SET analyses as well as the tree comparison analysis. BJ, LB, RO, and MW conceived the study and co-wrote the manuscript. All authors read and approved the final manuscript.

## Conflict of Interest Statement

BJ and LB are employed by company Nestlé Research. MW serves on paid scientific advisory groups for BioMerieux, Merieux NutriSciences, Neogen, Chris Hansen, Mars, and Thermo Fisher and has had paid speaking engagements for 3M. The remaining author declares that the research was conducted in the absence of any commercial or financial relationships that could be construed as a potential conflict of interest.

## Footnotes

1 http://www.bioinformatics.babraham.ac.uk/projects/fastqc/

## Supplementary Material

FIGURE S1 Phylogenetic trees and dendrograms obtained for each hqSNP and MLST pipeline for each cluster.

TABLE S1 Quality metrics for the 42 genomes sequenced here.

TABLE S2 Summary of the pairwise comparisons carried out using the hqSNP pipelines.
