# Supplementary figures and images for: Comparative Analysis of Tools and Approaches for Source Tracking Listeria monocytogenes in a Food Facility Using Whole-Genome Sequence Data
Source: Front Microbiol. 2019 May 9;10:947. doi: 10.3389/fmicb.2019.00947 (PMC6521219; doi:10.3389/fmicb.2019.00947)

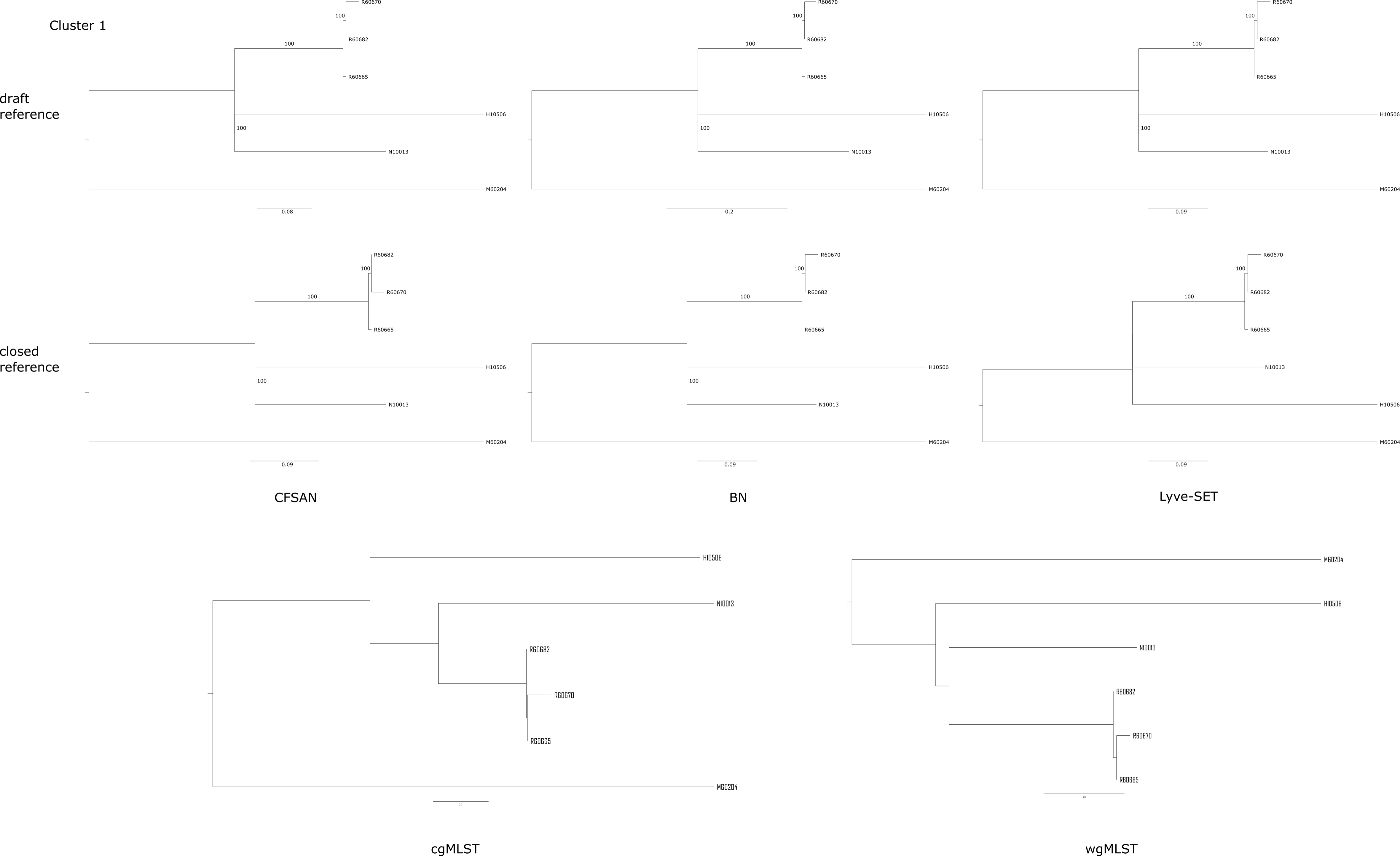

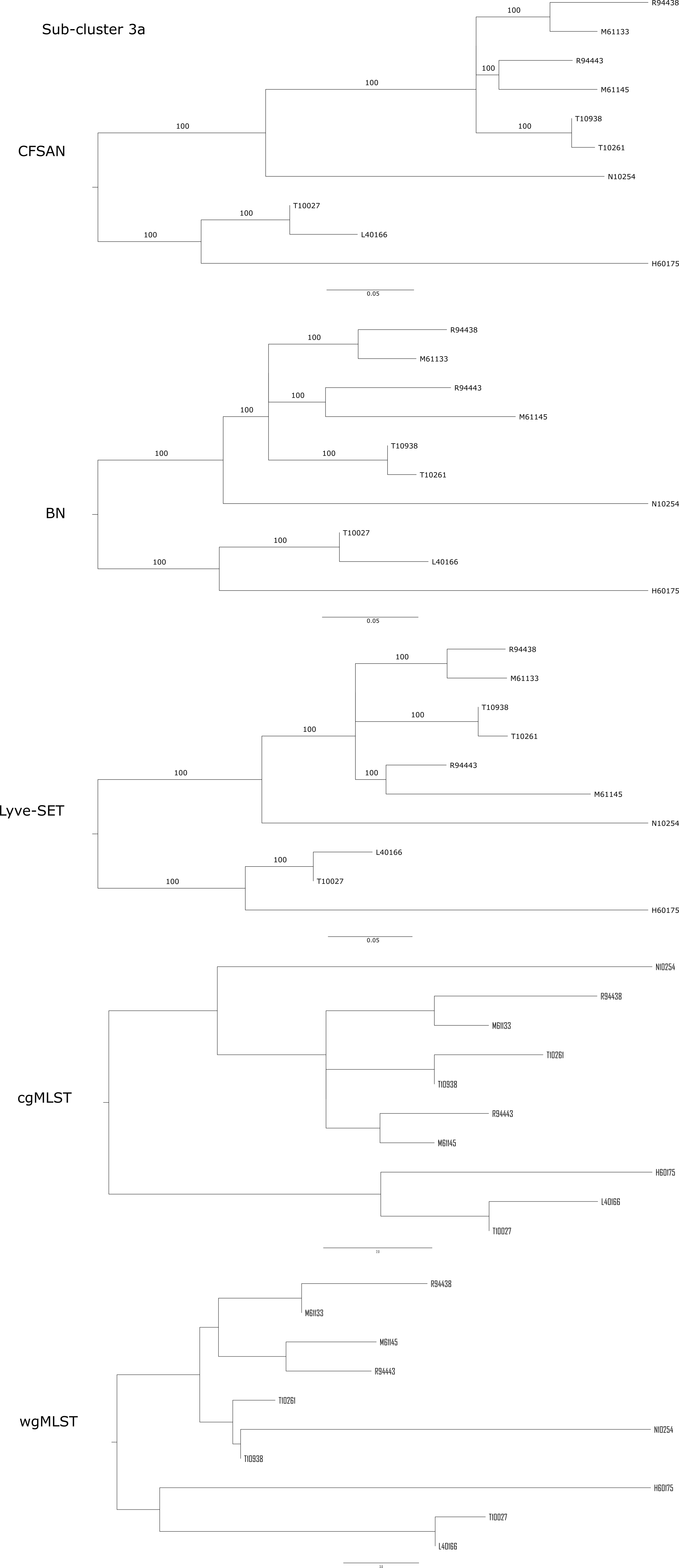

# CFSAN

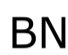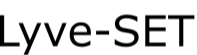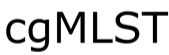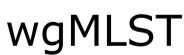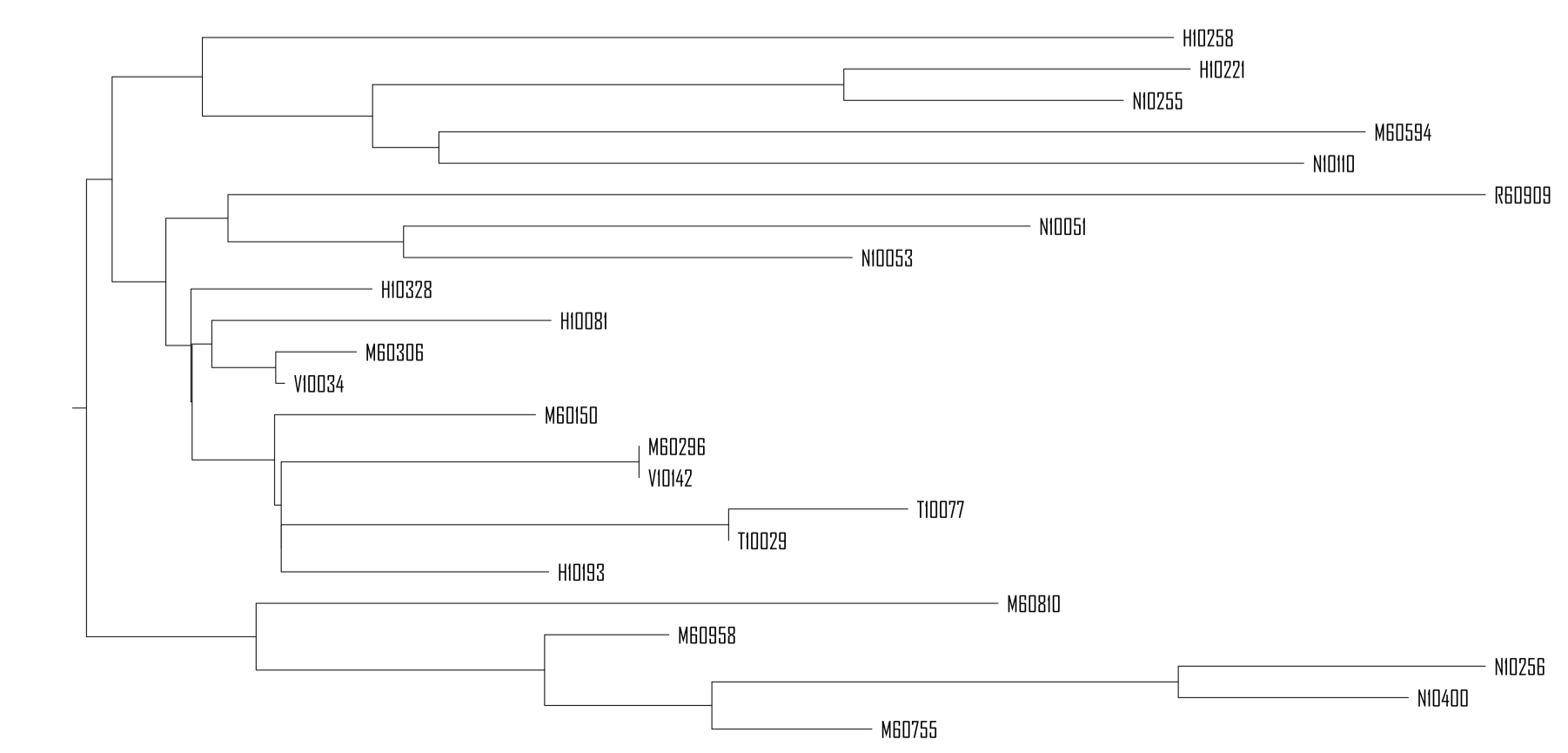

Supplement: FIGURE S1 — Phylogenetic trees and dendrograms obtained for each hqSNP and MLST pipeline for each cluster. [file Image_1.pdf]
